# Fanniidae (Diptera): new synonym, new records and an updated key to males of European species of *Fannia*

**DOI:** 10.3897/zookeys.593.7735

**Published:** 2016-05-26

**Authors:** Miroslav Barták, Jiří Preisler, Štěpán Kubík, Hana Šuláková, Vladislav Sloup

**Affiliations:** 1 Department of Zoology and Fisheries, Faculty of Agrobiology, Food and Natural Resources, Czech University of Life Sciences Prague, CZ-165 21 Praha 6-Suchdol, Czech Republic; 2 Vlnařská 692, CZ-460 01 Liberec 6, Czech Republic; 3 Institute of Criminalistics Prague, Czech Republic, P.O.Box 62/KUP, CZ-170 89 Prague 7

**Keywords:** Diptera, Calyptrata, Fanniidae, Europe, Czech Republic, Slovak Republic, Kazakhstan

## Abstract

Based on revision of large recent collections of the authors, the following five species are first recorded from the Czech Republic: *Fannia collini* d’Assis-Fonseca, 1966 (simultaneously first record in Central Europe), *Fannia lugubrina* (Zetterstedt, 1838), *Fannia melania* (Dufour, 1839), *Fannia slovaca* Gregor & Rozkošný, 2005, and *Fannia brinae* Albuquerque, 1951 (simultaneously first record from low altitudes). Another species, *Fannia alpina* Pont, 1970, is first recorded from Slovak Republic, and *Fannia cothurnata* (Loew, 1873) is first recorded from Kazakhstan. An updated key to males of European species of *Fannia* is presented. A list of Czech and Slovak Fanniidae is appended. One new synonym is established: *Fannia lucida* Chillcott, 1961 is considered junior synonym of *Fannia norvegica* Ringdahl, 1934. Altogether two species are first recorded from Bohemia [*Fannia cothurnata* (Loew, 1873) and *Fannia vespertilionis* Ringdahl, 1934] and three for Moravia [*Fannia alpina* Pont, 1970, *Fannia conspecta* Rudzinski, 2003, and *Fannia limbata* (Tiensuu, 1938) – this species considered in Central Europe very rare was found in numbers near waters both running and standing in early spring under unusually warm temperature conditions].

## Introduction

The Fanniidae are a small family of Calyptratae distributed worldwide, comprising more than 360 extant species (Pape et al. 2011) in 5 genera (*Euryomma*, *Fannia*, *Piezura*, *Australofannia*, *Zaelandofannia*). In Europe, 85 species are known (Pont 2007, Rudzinski 2003, Gregor and Rozkošný 2005). Some representatives are known from their forensic, medical and hygienic importance. Several species have a tendency for synanthropy. Females are attracted to decaying organic matter, often in great numbers. In addition, males are attracted to the same substrate but much less frequently. In our (unpublished) experiments with pig carcasses, almost 20 000 specimens were collected and females were about 13 times more frequent.

Adults may be distinguished from representatives of all other families of calyptrates by an asetose meron, the second anal vein strongly bent towards the first anal vein, so that prolongation of it will cross first anal vein at most at the wing margin, the scutellum without setulae on the lower surface, and the Sc vein having only one (basal) bend. Moreover, females lack crossed interfrontals and proclinate orbitals.

Larvae are aquatic to terrestrial, often living in semi-aquatic media. Larvae and puparium of fanniids are readily identifiable by sharing a dorso-ventrally flattened body, characterized by conspicuous feathery, forked, tufted, or button-like processes distributed over most of the dorsal and lateral surface of segments (and in reduced form also on ventral surface). An interesting character known at least in *Fannia canicularis* is a trichoid sensillum on the posterior spiracular plate, representing a sensory organ otherwise unknown in the Calyptratae (Grzywacz et al. 2012, Domínguez and Pont 2014).

For more details about morphology, biology, and zoogeography of the family see Chillcott (1961), Rozkošný et al. (1997), Pont (2000) or Domínguez and Pont (2014).

In the last 10–15 years, we collected some 200 000 specimens of Fanniidae mostly by means of mass collecting methods (Malaise traps, pyramidal traps exposed above pig carcass or heap of decaying wood, protein traps, yellow and white water pan traps, etc.) and stored them in ethyl alcohol. Using morphospecies method (based chiefly on examining male genitalia) we selected some 3 000 specimens which were dry mounted and identified to species. This revealed many important findings and the results of our studies are published herewith.

## Material and methods

This paper is based on extensive materials of Fanniidae deposited in the collection of the Czech University of Life Sciences, Prague (CULSP) and partly in the collection of the North Bohemian Museum, Liberec (NBML) and Institute of Criminalistics, Prague (ICP). Some specimens originate from the Canadian National collection of Insects and Arachnids, Ottawa (CNC), Natural History Museum, London
(NHM), National Museum, Prague (NMP) and Moravian Museum, Brno (MMB).

The identification of the Central European species is possible using the keys in the review of the European species (Rozkošný et al. 1997), which also summarises all the available data on the morphology of immature stages and adults, development and biology, medical, hygienic and economic importance, and distribution. More recently Pont (2002) has proposed some new synonyms based on a study of Zetterstedt´s types. Two recently described species, *Fannia conspecta*: Rudzinski (2003) and *Fannia slovaca*: Gregor and Rozkošný (2005), are lacking in the above mentioned keys. So we elaborated an updated key to males of European species of *Fannia*. In order to make our updated key more convenient for users, the couplets from Rozkošný et al. (1997) have ben maintained mostly unchanged, including reference to figures in that publication.

Distributional records are taken mainly from Pont (2007) if not stated otherwise.

Figure preparation: genitalia together with 2–3 pregenital segments were removed and macerated in potassium hydroxide solution (approx. 10%) in small vials submerged in hot water for 1–2 hours. After neutralizing with 8% acetic acid, the genitalia were dissected in glycerine and their parts (without hypandrium) photographed by means of an Olympus E-41 digital camera mounted on an Olympus BX51 compound microscope. Images were edited with the computer software Quick Foto micro 2.3 provided with Deep focus 3.1. Each image resulted usually from combining 7–15 layers. Images were improved by means of Adobe Photoshop.

Microphoto (Fig. 14) was preperaed by means of ZEISS Ultra Plus SEM operating at low accelerating voltage of 5 kV. A chamber secondary electron detector was used for imaging in topographical contrast. Before the analysis the sample was sputter-coated with 3 nm of platinum to obtain electrically conductive surface.

Abbreviations used: MT = Malaise trap, SW = sweeping, ET = emergence trap.

## Results

(species are arranged alphabetically)

*Fannia alpina* Pont, 1970. Material examined (2♂): 1♂, Slovakia b., V. Tatry Mts, Tatranská Lomnica - 3 km NW, 49°10'N, 20°15'E, 1100 m, 13.viii.1982, M. Barták; 1♂, Moravia bor., Beskydy, H. Lomná, Hruška, 49°30'29"N, 18°36'56"E, 23.v.-19.vi.1999, MT, M. Barták (- all CULSP). Broadly distributed (Palaearctic and Oriental region) but uncommon species, in Europe previously known from Austria, the Czech Republic and Finland. It has also been found in Japan (Nishida 1974) and Nepal (Nishida 1994). First record from Slovak Republic and Moravia.

*Fannia brinae* Albuquerque, 1951. Material examined: 1♂, Moravia mer., Hustopeče, 240 m, alfalfa, conventional agric., 45°57'39"N, 16°41'49"E, 1.-30.vii.2008, ET, J. Rotrekl (CULSP). Very rarely collected species known up to now only from a few localities in French Alps. Not only essential characters for recognition of the species (broad frons with developed orbitals and very long submedian anterodorsal and dorsal seta inserted close together on the same level) but also all other characters of the above specimen even in small details agree with redescription by Gregor and Rozkošný (1993) except the following: 7 pairs of strong orbitals present (with small hairs between them), uppermost one strong and lateroclinate and abdomen with narrow dark midline. The specimen possesses several characters mentioned in this species (and also in allied species, *Fannia altaica*) by Pont and Vikhrev (2009): several fine setulae present on upper part of parafacials (possibly a variable character), only a single small seta in addition to strong seta on both proepisternum and proepimeron and bare propleural depression, but contrary to this paper, fore tibia of our specimen has distinct (even if short) anterodorsal seta. Moreover, pedicel seems to be paler (reddish-brown) anteriorly near apex. First record for the Czech Republic and the first record from low altitudes.

**Figures 1–3.**
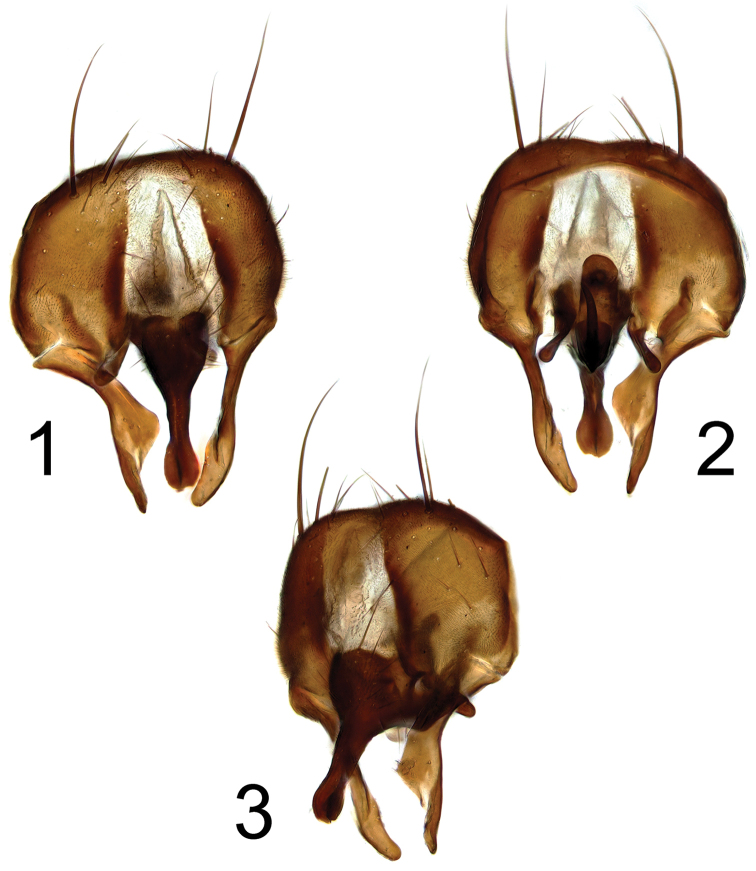
*Fannia carbonaria* (Meigen, 1826), hypopygium: **1** dorsal view **2** ventral view **3** oblique view.

*Fannia carbonaria* (Meigen, 1826) (Figs 1–3). Material examined (4♂): 1♂: Bohemia b., Krkonoše, Bíner, 609 m, damp meadow, 50°37'50"N, 14°40'34"E, 21.v.- 16.vi.2009, MT, J. Vaněk; 1♂: same data but 16.vi.-7.vii.2009 (- all CULSP); 1♂: Slovakia, Dvorčany, 16.iv.1957, J. Čepelák (MMB); 1♂: Kazakhstan, Almaty reg., Kazstroj, 1240 m, 43°17'26"N, 77°18'22"E, 7.-21.v.2013, MT, O. Nakládal (CULSP) – first record from Kazakhstan. Broadly distributed Holarctic species (also in Taiwan), but everywhere apparently rare. World species of *carbonaria* subgroup have been keyed by Wang et al. (2009), but mid tibia of *Fannia carbonaria*, stated here as having only 1 posterodorsal has in fact mostly at least 2 such setae (number varying from 1 to 5); also couplet 7 of their key is confusing because *Fannia carbonaria* has white squamae. Also in the key by Rozkošný et al. (1997) is this species wrongly arranged because it has no long posteroventrals at least on apical half of hind femur.

*Fannia collini* d’Assis-Fonseca, 1966. Material examined: 1♂, Bohemia b., Frýdlantská pahorkatina Hills, Poustecká obora nr. Poustka, 50°57'33.6"N, 15°3'50.9"E, 18.vii.-8.viii.2012, MT, J. Preisler & P. Vonička (NBML). The species has been known previously only from Great Britain. Our specimen agrees in nearly all details with original description incl. very distinctive genitalia. Slight differences are as follows: 12 pairs of orbital setae (and not „8-10“, as stated in the original description) and anterodorsal seta on t3 is very short and fine (and not „strong“). Males of *Fannia collini* may be easily identified using key in Rozkošný et al. (1997), female remains unknown. First record for the Czech Republic and in Central Europe.

*Fannia conspecta* Rudzinski, 2003. Material examined (10♂): Bohemia c., Praha Troja, 184 m, 50°7'15"N, 14°23'53"E, emergence trap baited with pig carcass, 1 ♂: 2.-9.v., 1♂: 15.-22.v., 2♂: 22.-29.v., 1♂10.-17.vii., 1♂: 17.-24.vii., 2♂: 4.-11.ix. - all 2012, M. Barták & H. Šuláková (CULSP); 1♂ Bohemia c., Mníšek pod Brdy, 8.viii.2012, 49°52'10"N, 14°15'38"E, 385 m, ex larva, from a human corpse, H. Šuláková; 1♂: Moravia, Hornomoravský úval, Kroměříž, nr. Moštěnka brook, 49°19'50"N, 17°23'10"E, 205 m, protein trap (chicken meat), H. Šuláková, 11.i.-20.iii.2010 (ICP). This species was known previously from the Czech Republic (Bílina – Jirásek III, 50°33'35"N, 13°47'44"E, 310 m, MT, Phragmitetum, 14.v.–23.vii.1998, M. Barták), Germany, Denmark, Portugal, Greece and South Russia (Grzywacz and Prado e Castro 2012). Additional records of this uncommon species from the Czech Republic were found and first records from Moravia.

*Fannia cothurnata* (Loew, 1873). Material examined: 1♂, Bohemia mer., Vráž nr. Písek, 400 m, nr. brook, 49°23'59"N, 14°7'58"E, 24.v.-24.vi.2010, MT, M. Barták; 1♂, Kazakhstan Almaty reg., Kazstroj, 1240 m, 43°17'26"N, 77°18'22"E, 7.-21.v.2013, MT, O. Nakládal (- all CULSP). Broadly distributed in Europe and Near East. In the Czech Republic published previously only from Moravia (Rozkošný and Gregor 1988). First records for Bohemia and Kazakhstan. The specimen from Kazakhstan has only one each antero- and posterodorsal seta on mid tibia but otherwise corresponds in all details to typical form.

*Fannia limbata* (Tiensuu, 1938). Material examined (13 ♂): 10♂: Moravia occ., Jihlava-Pávov, 495 m, 49°26'26"N, 15°35'44"E, wetland nr. pond, 16.iv.-3.v.2009, MT, M. Barták; 3♂: Bohemia b., Děčín-Čertova voda, right Labe shore, 130 m, 50°48'47"N, 14°13'35"E, MT baited with decaying meat, 11.-25.iv.2009, M. Barták (all CULSP). Rarely collected species known only from Scandinavia, Germany and the Czech Republic (previously one record only from Kostelní Lhota nr. Sadská). First record from Moravia and only the second from Bohemia. All Czech records originate from the vicinity of water (both running and standing) under unusually hot early spring conditions.

*Fannia lugubrina* (Zetterstedt, 1838). Material examined: 1♂, Bohemia b., Krkonoše Mts, Labská rokle nr. Labská bouda, 1300 m, 50°46'19"N, 15°32'43"E, 31.v.-15.vi.2007, MT, J. Vaněk (CULSP). A Holarctic species, in Europe distributed in Scandinavia and North Russia and several temperate European countries: Belgium, Austria, and Poland. First record for the Czech Republic.

*Fannia melania* (Dufour, 1839). Material examined: 2♂: Bohemia b., Jizerské hory Mts, Holubník Mt., Bílé Bukoví, 900 m, 50°49'57"N, 15°10'51"E, 16.vi.-14.vii.2011, MT, J. Preisler & P. Vonička (NBML, CULSP). Broadly distributed but apparently rare Eurasian species. First record for the Czech Republic.

**Figures 4–7.**
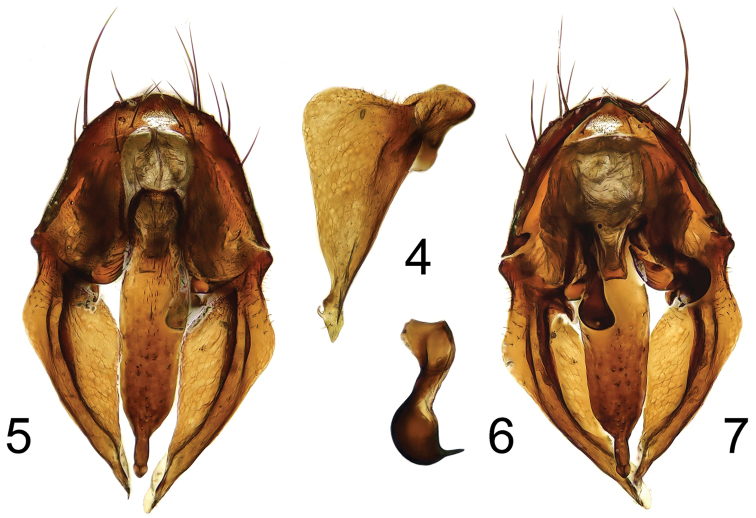
*Fannia nidica* Collin, 1939, hypopygium: **4** surstylus, standardized view (appearing broadest) **5** dorsal view **6** bacilliform scerite **7** ventral view.

*Fannia nidica* Collin, 1939 (Figs 4–7). Material examined (18♂): 2♂: Bohemia mer., Vráž nr. Písek, 400 m, nr. brook, 49°23'59"N, 14°7'58"E, 10.v.-4.vi.2011; 1♂: same locality, 2.iv.-10.v.2011; 1♂: same locality, 30.iv.-6.vi.2012; 1♂: Moravia, Jihlava-Pávov, wetland nr. pond, 495 m, 49°26'26"N, 15°35'44"E, 16.iv.-3.v.2009; 1♂: Bílina – Vršíček, 50°33'12"N, 13°49'57"E, 410 m, 30.iv.-13.v.1998, - all M. Barták (- all MT, CULSP); 10♂: Bohemia b., Frýdlantská pahorkatina Hills, Poustecká obora nr. Poustka, 50°57'34"N, 15°3'51"E, 27.iv.-16.v.2012, MT, J. Preisler & P. Vonička; 1♂: Bohemia b., Frýdlantská pahorkatina Hills, Černousy-V Poli nr. Dubový rybník, 50°59'46"N, 15°2'49"E, 16.v.-12.vi.2012, MT, J. Preisler & P. Vonička; 1♂: Bohemia b., Jizerské hory Mts, Šolcův rybník, env. Raspenava, 350 m, 50°52'49"N, 15°6'51"E, MT, 11.-26.v.2011, J. Preisler & P. Vonička (- all NBML). Very rare species, known only from England, Denmark and the Czech Republic (Sadská, Vršíček in NW Bohemia, and Podyjí NP – Gregor, Rozkošný, Barták & Kubík 2005). *Fannia nidica* has been erroneously placed in the key by Rozkošný et al. (1997) under couplet 31. However; it has usually 2 anterodorsal and 2 posterodosal setae on mid tibia (occasionally only 1 may be present on either side), which in fact leads the species to section 22. Moreover, *Fannia nidica* has relatively long (even if sparse) ommatrichia which may erroneously lead it to *Fannia hirticeps* in keys. However, the latter species has much narrower cercal plate, dark tip of halter and much shorter and broader midbasitarsal crest.

**Figures 8–10.**
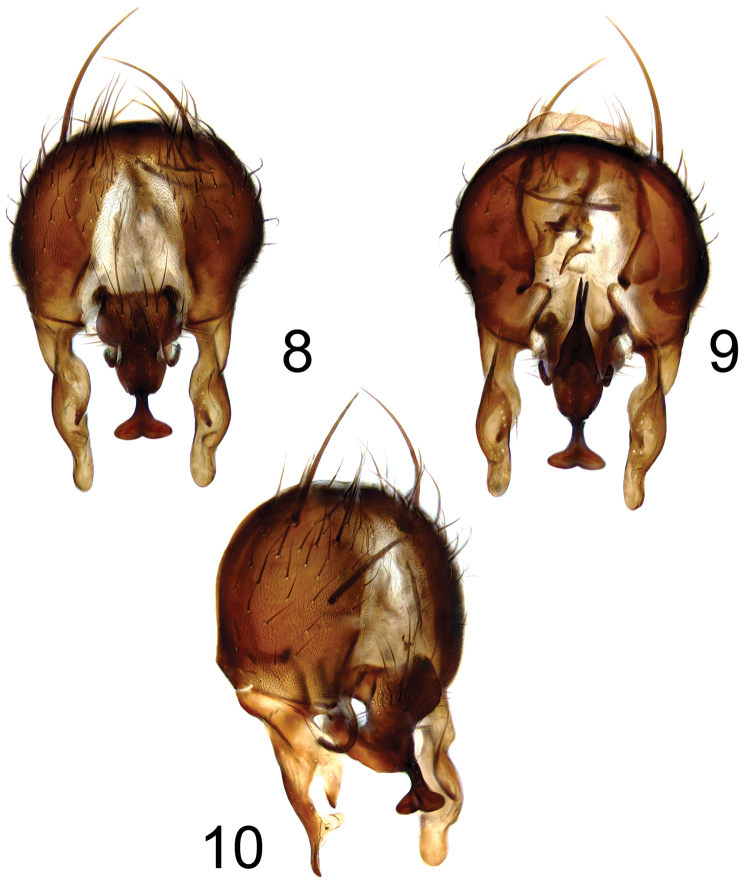
*Fannia norvegica* Ringdahl, 1934, hypopygium: **8** dorsal view **9** ventral view **10** oblique view.

*Fannia norvegica* Ringdahl, 1934 (Figs 8–10). Material examined (6♂): 1♂: Bohemia occ., Duchcov, 2 km E, willow shrubs, 50°37'N, 13°43'E, 240 m, 8.vii.1992, M. Barták; 2♂: Bohemia occ., Bílina, Choumek hill, 50°32'38"N, 13°51'32"E, 480 m, 24.vii.-24.viii.1998, MT, M. Barták; 1♂: Vráž nr. Písek, 400 m, 49°24'12"N, 14°7'3"E, 12.vi.-10.ix.2015, pyramidal trap with decaying wood, M. Barták (- all CULSP); 1♂: Mile 315 Alaska Richard. Hwy, 8.vi.1951 W. R. M. Mason (CNC – paratype of *Fannia lucida* Chillcott, 1961); 1♂: Wychwood Forest Oxon 1.vii.72, E. A. Fonseca, Pres. by E. C. M. Assis Fonseca BMNH 1988-212 (NHM). Broadly distributed, but uncommon species. Known from Norway, Spain, North Africa, G. Britain, Denmark, Greek, Switzerland and Japan. From the Czech Republic published from Bílina and Duchcov environs by Gregor and Barták (2001). *Fannia norvegica* was keyed by Wang et al. (2009) and they found it the closest to *Fannia lucida* Chillcott. It aroused our interest in the study of differences between these two species; moreover, cercal plate of our specimens seemed to be more similar to *Fannia lucida* (figured by Chillcott, 1961, fig. 74) than to *Fannia norvegica* (figured by d’Assis-Fonseca, 1968, fig. 37) especially by short “stem” before knob-like tip. Wang et al. (2009) stated differences between them as follows: “mid tibia with 2 ad; male cerci broad in distal half from ventral view, only apex slender“ - *Fannia lucida*, and: „mid tibia with 3 ad; male cerci distinctly slender in distal half from ventral view, slightly broadened at apex“ - *Fannia norvegica*. Ringdahl (1934) in original description also described mid tibia with 3 anterodorsals; however, Nishida (2003) redescribing *Fannia norvegica* stated: “mid tibia with 2 ad and 1-2 pd setae”. In the original description of *Fannia lucida* (Chillcott 1961), there is stated: „separable... from *norvegica* by the fewer tibial bristles“, but, their number is specified only in case of mid tibia: “two ads, two pds”. Collin (1958, Fig. 1a) noticed „projection X“ as a feature differing it from near *Fannia carbonaria* (beside presence of long posteroventrals on hind femur). To elucidate status of *Fannia lucida*, we borrowed one paratype specimen from CNC and found both species to be identical. The number of tibial setae is summarised in the Table 1. It seems clear that there is no difference between *Fannia lucida* and *Fannia norvegica* tibial setation.

**Table 1.** Setation of mid and hind tibia in all available specimens of *Fannia norvegica*.

	Mid tibia	Hind tibia
Paratype of *Fannia lucida*	2 ad (dorsal one short), 2 pd (dorsal shorter)	3 av (ventral very short), 2 ad, 2 d
Duchcov specimen	2 ad, 2 pd (all nearly equal in size)	3 av, 3–4 ad, 2 d
Vráž specimen	2 ad, 1–2 pd	2 av, 3 ad, 2 d
Chloumek specimen 1	2 ad (dorsal one shorter), 2 pd (nearly equal in size)	1–2 av, 2 ad, 2 d
Chloumek specimen 2	2 ad (dorsal one shorter), 2 pd (nearly equal in size)	1-2 av, 2–3 ad, 2 d
Wychwood specimen	2 ad, 2 pd	2 av, 2 ad, 2 d

Studying genitalia of both species we found them identical including basal outgrowth of surstyli (Collin´s 1958 “projection X” - Fig. 10), simply bent bacilliform sclerites and forked (V-shaped) tip of ventral part of cercal plate (Fig. 9). Thus, *Fannia lucida* Chillcott, 1961 is considered here junior synonym of *Fannia norvegica* Ringdahl, 1934. Interestingly, another species very similar to *Fannia norvegica* is *Fannia pseudonorvegica* d´Assis-Fonseca, 1966. The latter species differs only in details from *Fannia norvegica*, beside small crest on the base of mid basitarsus, basal process of surstyli seems to be larger (Fig. 13), apical broadening of cercal plate narrower (more linear than heart-shaped), and ventral process of cercal plate ends in two basally separated (U-shaped) processes (Fig. 12).

**Figures 11–13.**
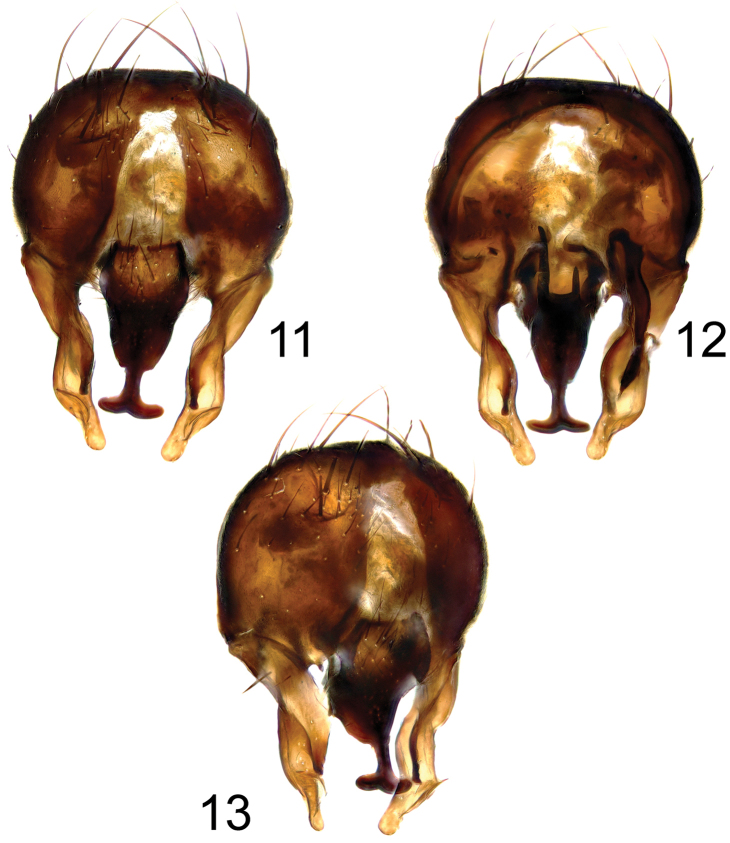
*Fannia pseudonorvegica* d´Assis-Fonseca, 1966, hypopygium: **11** dorsal view **12** ventral view **13** oblique view.

*Fannia slovaca* Gregor & Rozkošný, 2005. Material examined: 1♂, Bohemia occ., Bílina, Chloumek, hilltop steppe, 480 m, 50°32'38"N, 13°51'32"E, 25.vi.-24.vii.1998, MT, M. Barták (CULSP). Species recognized only recently, so its distribution is only poorly known, so far found only in Slovak Republic and Finland (Kahanpää and Haarto 2014). First record for the Czech Republic.

*Fannia verrallii* (Stein, 1895). Material examined (3♂): 1♂: Bohemia b., Krkonoše Mts, Labský důl nr. Labe river, 1040 m, 50°45'48"N, 15°33'05"E, 21.-28.vi.2006, MT, J. Vaněk; 1♂: Bohemia occ., Šumava Mts, Rokytecká slať peat-bog, 1100 m, 49°0'59"N, 13°25'5"E, 20.vii.-24.ix.1999, MT, M. Barták & Š. Kubík (- all CULSP); 1♂: Bohemia b., Jizerské hory Mts, Jizerka, 20.vi.2008, SW, J. Preisler (NBML). A rarely collected Holarctic species known in Europe only from G. Britain, Germany, Norway, Finland, Sweden, and the Czech Republic (Pont 2007). From Bohemia published by Gregor et al. (2003). Listed in Red list as vulnerable species in the Czech Republic (Gregor, Rozkošný and Barták 2005). Confirmed occurrence of this species in the Czech Republic and further records from Bohemia.

*Fannia vespertilionis* Ringdahl, 1934. 1♂: Bohemia c., Tiché údolí, Roztocký háj nr. Roztoky, 50°8'47.5"N, 14°23'10.1"E, 20.iv.-20.v.2009, beer trap, J. Preisler (NBML). Temperate European species. From the Czech Republic previously known only from Pálava BR (Gregor and Rozkošný 1999). Listed in Red list as vulnerable species in the Czech Republic (Gregor, Rozkošný and Barták 2005). First record from Bohemia.

### An updated key to males of European species of *Fannia*

(The male of *Fannia latifrontalis* Hennig is not known; all species included in Fauna Europea are keyed as well as all species described more recently.)

**Table:** 

1	Abdomen club-like, broadest just beyond middle (Rozkošný et al. 1997, Fig. 4q)	**2**
–	Abdomen normal, broadest in anterior half or at middle (Rozkošný et al. 1997, fig. 4r–t)	**3**
2 (1)	Lower margin of face distinctly produced, theca of proboscis longer than half length of fore tibia (Rozkošný et al. 1997, fig. 3c); abdomen entirely black; ventral parts of tergites 4 and 5 with long crossing setae (Rozkošný et al. 1997, fig. 4q) (terminalia: Rozkošný et al. 1997, fig. 11d)	***Fannia mollissima* (Haliday)**
–	Lower margin of face barely produced, theca of proboscis much shorter; abdomen with a yellow pattern in basal half; ventral part of tergites without crossing setae (terminalia: Rozkošný et al. 1997, fig. 16e)	***Fannia subpellucens* (Zetterstedt)**
3 (1)	Mid coxa with 1–3 strong hook-like setae; hind coxa with 1 or more setae on posterior inner margin (Rozkošný et al. 1997, fig. 4o); presutural acrostichal setulae triseriate	**4**
–	Mid coxa without strong hook-like setae	**12**
4 (3)	Mid coxa with 2–3 hook-Iike setae (Rozkošný et al. 1997, fig. 4o); mid tibia with a shining black inner projection (Rozkošný et al. 1997, fig. 4e) (terminalia: Rozkošný et al. 1997, fig. 13e)	***Fannia scalaris* (Fabricius)**
–	Mid coxa with 1 hook-like seta; mid tibia without inner projection	**5**
5 (4)	Fore tibia with a dense brush of flattened setae at apex laterally (Rozkošný et al. 1997, fig. 4m); fore coxa on lower inner margin with two grooved spines standing side by side (Fig. 14)	**6**
–	Fore tibia without a brush of flattened setae; fore coxa without spines on lower inner margin	**7**
6 (5)	Hind femur with strong anteroventral setae along almost whole length; hind tibia with a row of unequal posteroventral setae in apical 2/3; mid tibia remarkably dilated in apical half (terminalia: Rozkošný et al. 1997, fig. 10h)	***Fannia manicata* (Meigen)**
–	Hind femur only with 2–3 anteroventral setae before apex; hind tibia without posteroventral setae; mid tibia only slightly dilated in apical half (terminalia: Rozkošný et al. 1997, fig. 11e)	***Fannia monilis* (Haliday)**
7 (5)	Katepisternum with a straight spine on ventral side; at least hind tibia pale, yellow to reddish brown	**8**
–	Katepisternum without straight spine on ventral side; all tibiae predominantly black	**10**
8 (7)	Mid and hind femora yellow; hind tibia with a row of long fine anteroventral setae in apical 2/3, its ventral and posteroventral surface covered with dense short setae (terminalia: Rozkošný et al. 1997, fig. 10g)	***Fannia lustrator* (Harris)**
–	Mid and hind femora black; anteroventrals and ventral pubescence on hind tibia less conspicuous	**9**
9 (8)	Abdomen with a narrow undusted median stripe in posterior view; mid tibia only slightly dilated in apical half; hind tibia long and densely haired on ventral and posteroventral surfaces (terminalia: Rozkošný et al. 1997, fig. 8f)	***Fannia fuscula* (Fallén)**
–	Abdomen with a median row of trapezoid dark spots dilated towards hind margin of tergites; mid tibia remarkably dilated in apical half; hind tibia without long fine hairs (terminalia: Rozkošný et al. 1997, fig. 15d)	***Fannia vesparia* (Meade)**
10 (7)	Hind femur with only 4 strong anteroventral setae before apex; hind tibia with complete rows of long and fine anteroventral and anterodorsal setae; lower calypter brown, with almost black margin and fringe (terminalia:Rozkošný et al. 1997, fig. 11a)	***Fannia melania* (Dufour)**
–	Hind femur with a complete row of about 12 anteroventral setae; hind tibia at most with 8 anterodorsal and 6 anteroventral setae; lower calypter white, with yellowish margin and fringe	**11**
11 (10)	Palpi as broad as half width of flagellomere; several rows of setulae behind postocular setulae; fore tibia with a distinct anterodorsal seta (terminalia: Rozkošný et al. 1997, fig. 7a)	***Fannia atripes* (Stein)**
–	Palpi much less than half width of flagellomere; only one row of setulae behind postocular row; fore tibia without anterodorsal seta (terminalia: Rozkošný et al. 1997, fig.14c)	***Fannia subatripes* ď Assis-Fonseca**
12 (3)	Mid coxa with 2 short peg-like setae on outer side (Rozkošný et al. 1997, fig. 9d) (Finland)	***Fannia rabdionata* Karl**
–	Mid coxa without strong setae on outer side	**13**
13 (12)	Abdomen with a brown pattern on abdominal tergites 3 and 4 consisting of 2 pairs of round spots and a median vitta (cf. Rozkošný et al. 1997, fig. 4u)	**14**
–	Abdomen without a pattern of paired spots	**15**
14 (13)	Hind tibia with 1 anteroventral and 0 posteroventral seta; hind femur without a preapical ventral swelling, with the anteroventral setae only slightly longer than femoral depth and not curled at tips (terminalia: Rozkošný et al. 1997, fig. 10d)	***Fannia leucosticta* (Meigen)**
–	Hind tibia with numerous anteroventral and posteroventral setae; hind femur with a preapical ventral swelling bearing a number of long fine anteroventral setae that are longer than femoral depth and are curled at tips (terminalia: Rozkošný et al.1997, fig. 14h)	***Fannia pusio* (Wiedemann)**
15 (13)	Mid basal tarsomere with a crest (small spine- or toothlike process at extreme base ventrally) (Rozkošný et al. 1997, fig. 3p–r, 4j, 9e–f); inner posterior margin of hind coxa always bare	**16**
–	Mid basal tarsomere without crest; inner posterior margin of hind coxa with setae or bare	**33**
16 (15)	Fore basal tarsomere with brush-like hairs ventrally; cercal plate with long setae (Rozkošný et al.1997, fig. 7b)	***Fannia barbata* (Stein)**
–	Fore basal tarsomere without conspicuous ventral hairs; cercal plate with normal setae	**17**
17 (16)	Eyes haired, hairs at least as long as diameter of anterior ocellus	**18**
–	Eyes bare or with only very short and sparse hairs	**19**
18 (17)	Mid tibia with 2 anterodorsal and 2–3 posterodorsal setae; hind femur with dense hairlike antero- and posteroventral setae; hind tibia with a normaI preapical dorsal seta (terminalia: Rozkošný et al. 1997, fig. 9a)	***Fannia hirticeps* (Stein)**
–	Mid tibia only with 1 antero- and 1 posterodorsal seta; hind femur with 3–6 anteroventral and without posteroventral setae; hind tibia without dorsal preapical seta (terminalia: Rozkošný et al. 1997, fig. 12b) (Great Britain)	***Fannia novalis* Pont**
19 (17)	Mid tibia with a remarkable tubercle in basal half; body densely grey dusted (terminalia: Rozkošný et al. 1997, fig. 10a)	***Fannia krimensis* Ringdahl**
–	Mid tibia without a tubercle in basal half, at most slightly swollen; body less dusted	**20**
20 (19)	Mid tibia with 2–3 anterodorsal and 2 posterodorsal setae; hind femur with 3–4 anteroventral setae at apex	**21**
–	Mid tibia with 1 antero- and 1 posterodorsal seta; hind femur at most with 2 anteroventral setae at apex	**24**
21 (20)	Hind tibia clothed with long and dense ventral hairs and with several fine curled setae at apex (Rozkošný et al. 1997, fig. 4k; terminalia: Rozkošný et al. 1997, fig. 6f)	***Fannia armata* (Meigen)**
–	Hind tibia without long hairs and curled setae	**22**
22 (21)	Cercal plate narrowed apically (terminalia: Figs 4–7); hind tibia with one anteroventral and one anterodorsal seta; midbasitarsal crest very long (as long as or longer than diameter of mid tibia) and very narrow (only slightly broader than preapical setae); one preapical anterior and one preapical posterior seta on mid tibia	***Fannia nidica* Collin**
–	Cercal plate broadened apically; remaining characters different	**23**
23 (22)	Hind tibia with one anteroventral and one anterodorsal seta; postocular setulae uniserial (terminalia: Rozkošný et al. 1997, fig. 8c)	***Fannia cothurnata* (Loew)**
–	Hind tibia with 1–3 anteroventral and 2–3 anterodorsal setae; postocular setulae biserial (terminalia: figs 11–13)	***Fannia pseudonorvegica* d´Assis-Fonseca**
24 (20)	Hind femur without distinct anteroventral setae (terminalia: Rozkošný et al. 1997, fig. 13d); lower calypter strip-like	***Fannia rondanii* (Strobl)**
–	Hind femur with at least 1 strong anteroventral seta; lower calypter developed, lobe-like	**25**
25 (24)	Hind femur without posteroventral setae in apical half (terminalia: Rozkošný et al. 1997, fig. 10e)	***Fannia limbata* (Tiensuu)**
–	Hind femur with a row of posteroventral setae in apical half	**26**
26 (25)	Hind femur with 2–5 anteroventral setae before apex (terminalia: Rozkošný et al. 1997, fig. 13c)	***Fannia ringdahlana* Collin**
–	Hind femur with only 1 anteroventral seta before apex	**27**
27 (26)	Fore tibia with a row of elongate posteroventral hairs; cercal plate broad, deeply constricted before middle (Rozkošný et al. 1997, fig. 14a)	***Fannia spathiophora* Malloch**
–	Fore tibia without elongate posteroventral hairs; cercal plate without constriction before middle	**28**
28 (27)	Hind femur with 3–6 posteroventral setae	**29**
–	Hind femur with 7–14 posteroventral setae	**30**
29 (28)	Presutural acrostichal setulae triserial; ventral crest on mid basal tarsomere weak (Rozkošný et al. 1997, fig. 9e) (terminalia: Rozkošný et al. 1997, fig. 16a)	***Fannia aethiops* Malloch**
–	Presutural acrostichal setulae biserial; ventral crest on mid basal tarsomere well developed (Rozkošný et al. 1997, fig. 9f) (terminalia: Rozkošný et al. 1997, fig. 16d) (N Europe)	***Fannia stigi* Rognes**
30 (28)	Postocular setulae biserial; acrostichal setulae mainly triserial; mid tibia strongly flattened, with a posteroventral ridge in apical third (terminalia: Rozkošný et al. 1997, fig. 16c)	***Fannia bigelowi* Chillcott**
–	Postocular setulae uniserial; acrostichal setulae mainly biserial; mid tibia not strongly flattened	**31**
31 (30)	Scutum not pale dusted in front of scutellum, completely black; bacilliform process simply bent ventrally, long (Gregor and Rozkošný 2005, fig. 11)	***Fannia umbratica* Collin**
–	Scutum pale dusted in front of scutellum; bacilliform process spiralled, long or short	**32**
32 (31)	Ten to fifteen strong posteroventrals on hind femur (Gregor and Rozkošný 2005, fig. 9); bacilliform process short (Gregor and Rozkošný 2005, fig. 8)	***Fannia umbrosa* (Stein)**
–	Five strong posteroventrals on hind femur (Gregor and Rozkošný 2005, fig. 10); bacilliform process long (Gregor and Rozkošný 2005, fig. 7)	***Fannia slovaca* Gregor & Rozkošný**
33 (15)	Mid and hind femora yellow; abdomen with extensive yellow pattern or entirely reddish yellow	**34**
–	Mid and hind femora predominantly black; abdomen black, rarely with extensive yellow pattern	**36**
34 (33)	lnner posterior margin of hind coxa with 1 or more setae; abdomen including genitalia entirely reddish yellow (terminalia: Rozkošný et al. 1997, fig. 15e)	***Fannia vespertilionis* Ringdahl**
–	Inner posterior margin of hind coxa bare; abdomen dark with yellow pattern	**35**
35 (34)	Mid tibia with a remarkable tubercle in middle (Rozkošný et al. 1997, fig. 4a); hind tibia at apex and hind basal tarsomere long haired ventrally; lower calypter not projecting (terminalia: Rozkošný et al. 1997, fig. 12c)	***Fannia ornata* (Meigen)**
–	Mid tibia without median tubercle; hind leg without remarkable pubescence on tibia and basal tarsomere; lower calypter distinctly projecting (terminalia: Rozkošný et al. 1997, fig. 12h)	***Fannia posticata* (Meigen)**
36 (33)	Mid femur with a group of spine-Iike setae in middle (cf. Rozkošný et al. 1997, fig. 4a); hind tibia with only 1 dorsal seta, the preapical one absent (terminalia: Rozkošný et al. 1997, fig. 13h)	***Fannia sociella* (Zetterstedt)**
–	Mid femur without spine-like setae in middle; hind tibia always with 2 dorsal setae, median and preapical	**37**
37 (36)	Apex of abdomen globular; sternite 5 shining black, projecting downwards (terminalia: Rozkošný et al. 1997, fig. 8g, as *Fannia glaucescens*)	***Fannia lucidula* (Zetterstedt)**
–	Apex of abdomen not globular; sternite 5 dull and adpressed	**38**
38 (37)	Inner posterior margin of hind coxa with setae (Rozkošný et al. 1997, fig. 4f)	**39**
–	Inner posterior margin of hind coxa bare	**62**
39 (38)	Mid tibia with a conspicuous tubercle on inner surface (Rozkošný et al. 1997, fig. 4d)	**40**
–	Mid tibia without tubercle on inner surface	**41**
40 (39)	Tubercle on mid tibia below middle; presutural acrostichal setulae in 3–4 rows; 1 long and fine prealar seta; hind tibia with about 8 anteroventral setae (terminalia: Rozkošný et al. 1997, fig. 7h)	***Fannia coracina* (Loew)**
–	Tubercle on mid tibia above middle (Rozkošný et al. 1997, fig. 4d); presutural acrostichal setulae in 2 rows; 2 short prealar setae; hind tibia only with 1 anteroventral seta (terminalia: Rozkošný et al. 1997, fig. 14f)	***Fannia tuberculata* (Zetterstedt)**
41 (39)	Mid tibia along whole length with dense, short, uniform and erect ventral pubescence, about half as long as greatest diameter of tibia or shorter (Rozkošný et al. 1997, fig. 4b); presutural acrostichal setulae triserial	**42**
–	Mid tibia ventrally with sparser, not uniform and especially in apical half usually much longer hairs (Rozkošný et al. 1997, fig. 4c); presutural acrostichal setulae mostly biserial	**55**
42 (41)	Abdomen yellowish at least at base	**43**
–	Abdomen entirely black	**47**
43 (42)	Fronto-orbital plates separated by a narrow frontal vitta; mesonotum yellowish grey dusted, without longitudinal brown stripes; tibiae broadly yellow at bases (terminalia: Rozkošný et al. 1997, fig. 9b)	***Fannia hirundinis* Ringdahl**
–	Fronto-orbital plates touching at least in a short distance; mesonotum usually with conspicuous longitudinal stripes; at most fore tibia yellowish at base	**44**
44 (43)	Abdominal segments 2 and 3 predominantly yellow; black median vitta narrow, not dilated at posterior margin of tergites; scutum with 3 brown stripes (terminalia: Rozkošný et al. 1997, fig. 14b)	***Fannia speciosa* (Villeneuve)**
–	Abdominal segments 2 and 3 only with oval lateral yellow spots, black median vitta dilated towards posterior margin of tergites (Rozkošný et al. 1997, fig. 4r); scutum at most with 1 brown stripe	**45**
45 (44)	Several short setae distinct above anterodorsal seta on hind tibia; scutum with a median matt brown stripe (terminalia: Rozkošný et al. 1997, fig. 7c)	***Fannia canicularis* (Linnaeus)**
–	Without short setae above anterodorsal seta on hind tibia; scutum without median stripe	**46**
46 (45)	Proepisternal depression bare; hind femur shortly and densely haired on posteroventral surface; mid femur with short and dense antero- and posteroventral setae; prealar midway between suture and supra-alar seta (terminalia: Rozkošný et al. 1997, fig. 7f)	***Fannia clara* Collin**
–	Proepisternal depression with several small setulae; hind femur only with short and sparse fine hairs on posteroventral surface; setae on mid femur long and sparse; prealar closer to suture (terminalia: Rozkošný et al. 1997, fig. 8d)	***Fannia difficilis* (Stein)**
47 (42)	Hooked aedeagus present and usually exposed (Rudzinski 2003, fig. 2); surstyli broad and triangular in ventral view (Rudzinski 2003, fig. 4); proepimeral seta with two or more adjacent setulae; proepisternal depression without setae; hind tibia with 1–2 anteroventrals and no posteroventral	***Fannia conspecta* Rudzinski**
–	Aedeagus membranose; main process of surstyli narrow and parallel-sided; remaining characters different	**48**
48 (47)	Palpi dilated and flattened, almost as broad as antennal flagellomere (Rozkošný et al. 1997, fig. 3g); mid femur with several rows of strong setae on posteroventral surface (terminalia: Rozkošný et al. 1997, fig. 10b)	***Fannia latipalpis* (Stein)**
–	Palpi not dilated and flattened; mid femur with uniserial (or exceptionally with 2–3 rows of) posteroventral setae	**49**
49 (48)	Distance between eye margins about twice as broad as antennal flagellomere; hind tibia with strong anterodorsal and dorsal setae at about same Ievel (terminalia: Rozkošný et al. 1997, fig. 9c)	***Fannia brinae* Albuquerque**
–	Distance between eye margins much narrower; anterodorsal and dorsal setae on hind tibia inserted at different levels	**50**
50 (49)	Scutum with 2 longitudinal brown stripes; postocular setulae biserial; hind tibia with 5–7 posteroventral setae (terminalia: Rozkošný et al. 1997, fig. 9i)	***Fannia incisurata* (Zetterstedt)**
–	Scutum with 1 or 3 longitudinal brown stripes or completely black; postocular setulae uniserial; hind tibia without posteroventral setae	**51**
51 (50)	Proepimeral seta surrounded by several setulae	**52**
–	Proepimeral seta with only 1 adjacent setula	**53**
52 (51)	Proepisternal depression with a few setulae; hind tibia with 1–2 anteroventral setae; hind femur with short posteroventral setae which are not as long as femoral depth (terminalia: Rozkošný et al. 1997, fig. 14g)	***Fannia monticola* Pont**
–	Proepisternal depression bare; hind tibia with 2–5 anteroventral setae; hind femur with posteroventral setae that are much longer than femoral depth (terminalia: Rozkošný et al. 1997, fig. 6d)	***Fannia aequilineata* Ringdahl**
53 (51)	Squamae with brown margin; mesoscutum deep black; abdomen with bluish shine (Canary Islands)	***Fannia pubescens* Stein**
–	Squamae without brown margin; mesoscutum light grey; abdomen without bluish shine	**54**
54 (53)	Hind tibia with 2 equally strong anteroventral setae; scutum with a median brown longitudinal stripe (dark form; see 44)	***Fannia canicularis* (Linnaeus)**
–	Hind tibia usually with 1 anteroventral seta; if 2 developed, then upper obviously shorter; scutum brownish black, without median stripe (terminalia: Rozkošný et al. 1997, fig. 14d)	***Fannia subpubescens* Collin**
55 (41)	Mid tibia with 2 or more antero- and posterodorsal setae (Rozkošný et al. 1997, fig. 4c)	**56**
–	Mid tibia only with 1 antero- and 1 posterodorsal seta	**59**
56 (55)	Hind femur in apical third with a tubercle bearing 12–15 posteroventral setae (terminalia: Rozkošný et al. 1997, fig. 16b)	***Fannia lugubrina* (Zetterstedt)**
–	Hind femur in apical third without tubercle	**57**
57 (56)	Ventral hairs on mid tibia not longer than greatest diameter of tibia; palpi shorter than half length of theca (Rozkošný et al. 1997, fig. 3e) (terminalia: Rozkošný et al. 1997, fig. 11c)	***Fannia minutipalpis* (Stein)**
–	At least some ventral hairs on mid tibia longer than greatest diameter of tibia (Rozkošný et al. 1997, fig. 4c); palpi longer than half length of theca (Rozkošný et al. 1997, fig. 3d)	**58**
58 (57)	Hind tibia with 3–4 anteroventral setae; longest ventral hairs on mid tibia about 1.5 times longer than greatest diameter of tibia (fig 4c) (terminalia: Rozkošný et al. 1997, fig. 12f)	***Fannia polychaeta* (Stein)**
–	Hind tibia with only 1–2 anteroventrals; ventral hairs on mid tibia shorter though overreaching diameter of tibia (terminalia: Rozkošný et al. 1997, fig. 11h)	***Fannia pauli* Pont**
59 (55)	Prealar seta completely absent; presutural acrostichal setulae always biserial (terminalia: Rozkošný et al. 1997, fig. 5a–e)	***Fannia genualis* (Stein)**
–	One or two prealar setae present; presutural acrostichal setulae in 2 or 3 rows	**60**
60 (59)	Hind tibia without posteroventral setae; abdomen with a narrow median vitta which may be absent on tergite 5 (terminalia: Rozkošný et al. 1997, fig. 7g)	***Fannia collini* ď Assis-Fonseca**
–	Hind tibia with a distinct row of posteroventral setae; median spots on abdomen remarkably dilated towards posterior margin of tergites	**61**
61 (60)	Posteroventral setae on hind tibiae longer than anterodorsal setae (Rozkošný et al. 1997, fig. 4l); hind femur with a complete row of anteroventral setae, distal 4–5 of them stronger (terminalia: Rozkošný et al. 1997, fig. 9h)	***Fannia immutica* Collin**
–	Posteroventral and anterodorsal setae on hind tibia of the same length; hind femur with only 2 anteroventral setae (terminalia: Rozkošný et al. 1997, Fig. 10c)	***Fannia lepida* (Wiedemann)**
62 (38)	Upper half of parafacials with a row of short setulae (terminalia: Rozkošný et al. 1997, fig. 10f)	***Fannia lineata* (Stein)**
–	Parafacials bare, rarely with a few isolated setulae	**63**
63 (62)	Lower calypter very narrow, strip-like, narrower than 1/3 of upper calypter (Rozkošný et al. 1997, fig. 3m–o)	**64**
–	Lower calypter rounded, broader than 1/2 of upper calypter (cf. Rozkošný et al. 1997, fig. 3k–l)	**71**
64 (63)	Mid and hind tibiae reddish brown to yellow	**65**
–	AlI tibiae mainly black	**66**
65 (64)	Thorax and abdomen mainly black; abdomen with a median row of subtriangular spots (terminalia: Rozkošný et al. 1997, fig. 12d)	***Fannia pallitibia* (Rondani)**
–	Thorax and abdomen densely grey dusted; abdomen with a narrow median vitta (terminalia: Rozkošný et al. 1997, fig. 13a)	***Fannia pruinosa* (Meigen)**
66 (64)	Hind femur with 3–4 posteroventral setae in apical half equalling greatest width of femur	**67**
–	Hind femur without elongate posteroventral setae	**68**
67 (66)	Presutural acrostichal setulae triserial; cercal plate tapered in distal part (rozkošný et al. 1997, Fig. 6e)	***Fannia alpina* Pont**
–	Presutural acrostichal setulae biserial; cercal plate distally T-shaped dilated (Rozkošný et al. 1997, fig. 7e)	***Fannia carbonella* (Stein)**
68 (66)	Parafacials indistinct in lateral view; cercal plate with two rounded processes (terminalia: Rozkošný et al. 1997, fig. 12e)	***Fannia parva* (Stein)**
–	Parafacials distinct in lateral view; cercal plate flat and dilated, without two rounded processes	**69**
69 (68)	Upper postocular setulae uniserial and uniform in length; mostly only one strong prealar seta; abdomen with a narrow median stripe on tergites 4 and 5 (terminalia: Rozkošný et al. 1997, fig. 13g)	***Fannia similis* (Stein)**
–	Upper postocular setulae biserial or at least alternating long and much shorter ones; usually two prealar setae; abdomen with a narrow median stripe on tergites 4 and 5 or with dark spots dilated posteriorly	**70**
70 (69)	Abdomen with a dark median stripe of uniform width; fore tibia yellowish basally (terminalia: Rozkošný et al. 1997, fig. 14e)	***Fannia subsimilis* Ringdahl**
–	Abdomen with dark spots dilated towards posterior margin of tergites; fore tibia usually dark basally (terminalia: Rozkošný et al. 1997, fig. 13f)	***Fannia serena* (Fallén)**
71 (63)	Hind femur with a preapical tubercle bearing a cluster of dense setae (Rozkošný et al. 1997, fig. 4g, h)	**72**
–	Hind femur without setose tubercle	**73**
72 (71)	Hind femur strongly curved (Rozkošný et al. 1997, fig. 4g); abdomen yellowish at base (terminalia: Rozkošný et al. 1997, fig. 8e)	***Fannia fasciculata* (Loew)**
–	Hind femur not curved (Rozkošný et al. 1997, fig. 4h); abdomen entirely dark (terminalia: Rozkošný et al. 1997, fig. 11 b)	***Fannia metallipennis* (Zetterstedt)**
73 (71)	Mid tibia with only 1 anterodorsal seta	**74**
–	Mid tibia at least with 2 anterodorsal setae	**79**
74 (73)	Hind tibia with 3–4 anterodorsal and at least 2 anteroventral setae	**75**
–	Hind tibia with only 1 anterodorsal and 1 anteroventral seta	**76**
75 (74)	Hind tibia with about 10 long and fine anteroventral and numerous hairlike ventral and posteroventral setae (terminalia: Rozkošný et al. 1997, fig. 16f) (Balearics, N Africa)	***Fannia tunisiae* Chillcott**
–	Hind tibia only with 2 anteroventral and without elongate ventral and posteroventral setae (terminalia: Rozkošný et al. 1997, fig. 11g)	***Fannia nigra* Malloch**
76 (74)	Abdomen yellowish at base; cercal plate about 5 times longer than broad (terminalia: Rozkošný et al. 1997, fig. 8h)	***Fannia gotlandica* Ringdahl**
–	Abdomen entirely black; cercal plate broader	**77**
77 (76)	Hind femur with fine, long and curled posteroventral setae in basal half (terminalia: Rozkošný et al. 1997, fig. 15c)	***Fannia verrallii* (Stein)**
–	Hind femur in basal half with posteroventrals at most half as long as depth of femur	**78**
78 (77)	Hind femur with one strong anteroventral seta (terminalia: Rozkošný et al. 1997, fig. 16a) (form without ventral crest on mid basal tarsomere; see 29)	***Fannia aethiops* Malloch**
–	Hind femur with 5–11 anteroventral setae (terminalia: Rozkošný et al. 1997, fig. 12g)	***Fannia postica* (Stein)**
79 (73)	Hind femur with long setae on ventral and posterior surface subapically, the longest of these about as long as half length of hind tibia (terminalia: Rozkošný et al. 1997, fig. 6g)	***Fannia atra* (Stein)**
–	Hind femur without such long setae in apical half	**80**
80 (79)	Halter dark apically; cercal plate about as long as broad, with two short divergent apical processes (terminalia: Rozkošný et al. 1997, fig. 8b); upper postoculars long and unequal in length (alternating long and short setae) and partly biserial	***Fannia corvina* (Verrall)**
–	Halter clear yellow; cercal plate different; upper postoculars equally short and uniserial (except *Fannia carbonaria*)	**81**
81 (80)	Hind femur with complete row of long posteroventrals longer than depth of femur in apical part; surstylus with basal outgrowth (terminalia: Figs 8–10)	***Fannia norvegica* Ringdahl**
–	Hind femur without posteroventrals or at most with short posteroventrals on basal part; surstylus without basal outgrowth	**82**
82 (81)	Cercal plate with two bowed ribs but without apical projection (terminalia: Rozkošný et al. 1997, fig. 8a)	***Fannia fuscitibia* Stein**
–	Cercal plate with apical projection button-like broadened apically but without two bowed ribs (terminalia: Figs 1–3)	***Fannia carbonaria* (Meigen)**

**Figure 14.**
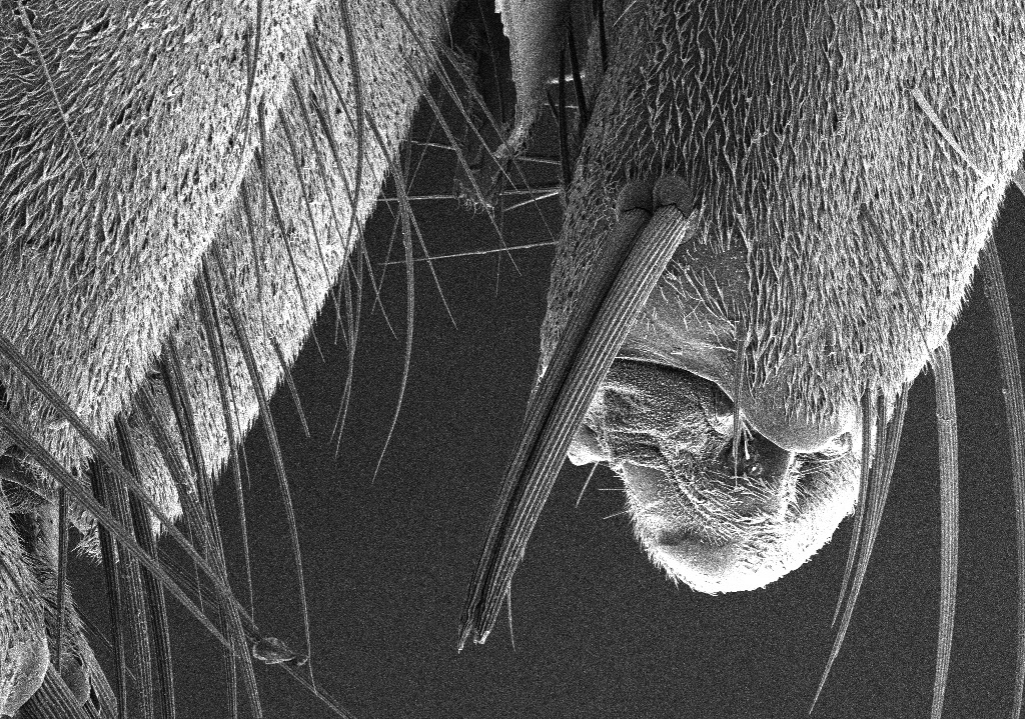
*Fannia manicata* (Meigen, 1826), two grooved spines standing side by side on fore coxa.

### Checklist of Czech and Slovak species

The last checklist of Czech and Slovak Fanniidae (Gregor and Rozkošný et al. 1997, 2009) contains 66 species: 64 from the Czech Republic (60 from Bohemia and 60 from Moravia) and 50 from Slovakia. Recently, *Fannia conspecta* and *Fannia latifrontalis* were published from the Czech Republic (Grzywacz and Prado e Castro 2012 and Preisler et al. 2013, respectively), which, together with 5 species first recorded herewith raised the number of known Czech species to 71. Slovak species are less known, two species have been added to last checklist (*Fannia tuberculata* and *Fannia speciosa*: Straka 2011) and another is added herewith raising the total number of known Slovak species to 53. All species previously published from the Czech Republic but not present in CULSP or NBML but deposited in NMP or MMB were checked to avoid the inclusion of questionable species.

[Arranged according to tradition of Czech and Slovak checklists of Diptera: Ježek (ed.) (1987), Chvála (ed.) (1997), Jedlička et al. (eds) (2006, 2009)]. B = Bohemia, M = Moravia, SK = Slovakia. All additions to current checklist (Gregor and Rozkošný et al. 1997, 2009) are signed with *.

***Piezura* Rondani, 1866**

*graminicola* (Zetterstedt, 1846) (B, M), SK

*pardalina* Rondani, 1866 (B, M) SK

***Fannia* Robineau-Desvoidy, 1830**

*aequilineata* Ringdahl, 1945 (B, M), SK

*armata* (Meigen, 1826) (B, M), SK

*alpina* Pont, 1970 (B, M*), SK*

*atra* (Stein, 1895) (B, M), SK

*atripes* Stein, 1916 (B, M)

*barbata* (Stein, 1892) (B, M), SK

*brinae* Albuquerque, 1951 M*

*canicularis* (Linnaeus, 1761) (B, M), SK

*carbonaria* (Meigen, 1826) (B, M), SK

*carbonella* (Stein, 1895) (B, M), SK

*clara* Collin, 1939 (B, M)

*collini* d’Assis-Fonseca, 1966 (B*)

*conspecta* Rudzinski, 2003 (B*, M*)

*coracina* (Loew, 1873) (B, M), SK

*corvina* (Verrall, 1892) (B, M), SK

*cothurnata* (Loew, 1873) (B*, M), SK

*difficilis* (Stein, 1895) (B, M), SK

*fasciculata* (Loew, 1873) (M)

*fuscitibia* Stein, 1920 (B, M)

*fuscula* (Fallén, 1825) (B, M), SK

*genualis* (Stein, 1895) (B, M), SK

*hirticeps* (Stein, 1892) (B, M), SK

*immutica* Collin, 1939 (B, M), SK

*incisurata* (Zetterstet, 1838) (B, M), SK

*krimensis* Ringdahl, 1934 (M), SK

*latifrontalis* Hennig, 1955 (B*)

*latipalpis* (Stein, 1892) (B, M), SK

*lepida* (Wiedemann, 1817) (B, M), SK

*leucosticta* (Meigen, 1838) (B, M), SK

*limbata* (Tiensuu, 1938) (B, M*)

*lineata* (Stein, 1895) (B, M)

*lucidula* (Zetterstedt, 1860) (B, M), SK

*lugubrina* (Zetterstedt, 1838) (B*)

*lustrator* (Harris, 1780) (B, M), SK

*manicata* (Meigen, 1826) (B, M), SK

*melania* (Dufour, 1839) (B*), SK

*metallipennis* (Zetterstedt, 1838) (B, M), SK

*minutipalpis* (Stein, 1895) (B, M), SK

*mollissima* (Haliday in Westwood, 1840) (B, M), SK

*monilis* (Haliday, 1838) (B, M), SK

*nidica* Collin, 1939 (B, M)

*nigra* Malloch, 1910 (B, M)

*norvegica* Ringdahl, 1934 (B)

*ornata* (Meigen, 1826) (B, M), SK

*pallitibia* (Rondani, 1866) (B, M), SK

*parva* (Stein, 1895) (B, M), SK

*pauli* Pont in Rozkošný, Gregor & Pont, 1997 (B, M), SK

*polychaeta* (Stein, 1895) (B, M), SK

*postica* (Stein, 1895) (B, M), SK

*posticata* (Meigen, 1826) (B, M), SK

*pruinosa* (Meigen, 1826) (B, M), SK

*pseudonorvegica* d´Assis-Fonseca, 1966 (B)

*ringdahlana* Collin, 1939 (B, M), SK

*rondanii* (Strobl, 1893) (B, M), SK

*scalaris* (Fabricius, 1794) (B, M), SK

*serena* (Fallén, 1825) (B, M), SK

*similis* (Stein, 1895) (B, M), SK

*slovaca* Gregor & Rozkošný, 2005 (B*) SK

*sociella* (Zetterstedt, 1845) (B, M), SK

*spathiophora* Malloch, 1918 (B, M)

*speciosa* (Villeneuve, 1898) (B, M) SK*

*subpubescens* Collin, 1958 (B, M), SK

*subsimilis* Ringdahl, 1934 (B, M), SK

*tuberculata* (Zetterstedt, 1849) (B, M), SK*

*umbratica* Collin, 1939 (B, M), SK

*umbrosa* (Stein, 1895) (B, M), SK

*verrallii* (Stein, 1895) (B, M)

*vesparia* (Meade, 1891) (B, M), SK

*vespertilionis* Ringdahl, 1934 (B*, M)

## Discussion

There are three important records of Central European Fanniidae that have mostly been overlooked because they were published in small local proceedings or journals:

*Fannia speciosa*: Eurasian species, recorded from Japan by Nishida (1976). In spite of being considered rare in central Europe (Rozkošný et al. 1997), we found surprisingly large numbers of specimens in Vráž near Písek (some 500 specimens, mostly females), especially from a pyramidal trap inserted above a large heap of decaying wood (see Preisler et al. 2013). From Slovakia reported only recently by Straka (2011).

*Fannia latifrontalis*: from the Czech Republic known only from a single female taken in Vráž near Písek (Preisler et al. 2013). For further comments about this seemingly very rare species see Kahanpää and Haarto (2014).

*Fannia tuberculata*: another rare species known previously from only two Czech Republic records: Mariánské Lázně and Lačnov near. Valašské Klobúky (see Rozkošný and Gregor 1988). From Slovak Republic reported by Straka (2011).
